# γ-Tocotrienol induces apoptosis in pancreatic cancer cells by upregulation of ceramide synthesis and modulation of sphingolipid transport

**DOI:** 10.1186/s12885-018-4462-y

**Published:** 2018-05-16

**Authors:** Victoria E. Palau, Kanishka Chakraborty, Daniel Wann, Janet Lightner, Keely Hilton, Marianne Brannon, William Stone, Koyamangalath Krishnan

**Affiliations:** 1 Division of Hematology-Oncology, Department of Internal Medicine, James H. Quillen College of Medicine, East Tennessee State University, Johnson City, TN 37614 USA; 2 Department of Pharmaceutical Sciences, Gatton College of Pharmacy, East Tennessee State University, Johnson City, TN 37614 USA; 3 Department of Internal Medicine, Beth Israel Deaconess Medical Center, Harvard Medical School, Boston, MA 02215 USA; 4 Department of Pediatrics, James H. Quillen College of Medicine, East Tennessee State University, Johnson City, TN 37614 USA

**Keywords:** γ-Tocotrienol, Vitamin E, Pancreatic cancer, ARV-1, CERT, Ceramide transport, Ceramide synthesis, Ceramide distribution, Lipid transport, Membrane lipid, Free radicals

## Abstract

**Background:**

Ceramide synthesis and metabolism is a promising target in cancer drug development. γ-tocotrienol (GT3), a member of the vitamin E family, orchestrates multiple effects that ensure the induction of apoptosis in both, wild-type and RAS-mutated pancreatic cancer cells. Here, we investigated whether these effects involve changes in ceramide synthesis and transport.

**Methods:**

The effects of GT3 on the synthesis of ceramide via the *de novo* pathway, and the hydrolysis of sphingomyelin were analyzed by the expression levels of the enzymes serine palmitoyl transferase, ceramide synthase-6, and dihydroceramide desaturase, and acid sphingomyelinase in wild-type RAS BxPC3, and RAS-mutated MIA PaCa-2 and Panc 1 pancreatic cancer cells. Quantitative changes in ceramides, dihydroceramides, and sphingomyelin at the cell membrane were detected by LCMS. Modulation of ceramide transport by GT3 was studied by immunochemistry of CERT and ARV-1, and the subsequent effects at the cell membrane was analyzed via immunofluorescence of ceramide, caveolin, and DR5.

**Results:**

GT3 favors the upregulation of ceramide by stimulating synthesis at the ER and the plasma membrane. Additionally, the conversion of newly synthesized ceramide to sphingomyelin and glucosylceramide at the Golgi is prevented by the inhibition of CERT. Modulation ARV1 and previously observed inhibition of the HMG-CoA pathway, contribute to changes in membrane structure and signaling functions, allows the clustering of DR5, effectively initiating apoptosis.

**Conclusions:**

Our results suggest that GT3 targets ceramide synthesis and transport, and that the upregulation of ceramide and modulation of transporters CERT and ARV1 are important contributors to the apoptotic properties demonstrated by GT3 in pancreatic cancer cells.

## Background

Pancreatic cancer is the fourth leading cause of cancer-related deaths in the United States [1]. Since available treatment options are limited, novel therapeutic agents that demonstrate the ability to inhibit signaling pathways implicated in the proliferation and survival of pancreatic cancer cells need to be evaluated for drug development. Promising agents may uncover new targets and alternate treatment strategies that have the potential to contribute to the understanding of pancreatic carcinogenesis and progression. It has been known for a long time that ceramides can inhibit cell proliferation and induce apoptosis in cancer cells via various stress stimuli such as tumor necrosis factor-α and platelet-activating factor [2, 3]. Furthermore, recent studies in the synthesis and metabolism of ceramides suggest that changes in the expression levels of these compounds may contribute to metastasis and resistance to therapy [4]. The syntheses of ceramides occur through multiple pathways that include: *de novo* synthesis from serine and palmitoyl-CoA substrates, salvage, from sphingosine [5] and from the hydrolysis of sphingomyelin by acid sphingomyelinase (ASM). The *de novo* synthesis is initiated in the cytoplasmic face of the endoplasmic reticulum by serine palmitoyl transferase (SPT), to form 3-keto-sphinganine, which is subsequently reduced to sphinganine (SA). Ceramide synthase (CerS) acetylates SA followed by desaturation by ceramide desaturase (DES) to form ceramide [6, 7]. There are six CerSs that regulate ceramide synthesis to produce a variety of compounds with di-and tri-hydroxy long-chain bases linked to fatty acids of variable length [8] and with C16 and C24 ceramides being most abundant in mammalian cells. These highly hydrophobic molecules can displace cholesterol and disrupt lipids rafts that may be associated with signaling molecules, thus affecting their function [9]. Moreover, the biophysical properties of ceramides may influence lipid reorganization in the membrane and cause destabilization, efflux and fusion. Hence, their expression levels and localization are tightly controlled.

Tocotrienols are members of the vitamin E family that unlike tocopherols possess an unsaturated isoprenoid side-chain [10]. These compounds have shown cytotoxic activity on pancreatic cancer cells via a multi-pronged mechanism. We had previously shown that γ-tocotrienol (GT3) is cytotoxic to pancreatic cancer cells, and is significantly more potent in its ability to inhibit cell viability as compared to alpha-tocopherol [11]. The ability of tocotrienols to selectively inhibit the PI3 kinase/Akt pathway, Ras/Raf/Erk signaling [11], HMG CoA reductase, and transcription factor NF-κB [12], are contributors to these properties. In pancreatic cancer, the oncogenic process is frequently driven by aberrant K-Ras. We have shown that GT3 can cause inhibition of cellular proliferation and survival in pancreatic cancer cells regardless of their K-Ras status [11]. However, the mechanism of action is not completely understood. It has been reported that vitamin E isoforms other than tocotrienols can increase cellular ceramide and dihydroceramide levels. Alpha-TEA, a modified form of alpha tocopherol, can increase membrane ceramide levels in mammary cancer cells [13], and γ-tocopherol has a similar effect on prostate cancer cells [14]. In vivo, pharmacokinetics studies have demonstrated the bioavailabilty of tocotrienols in humans [15]. These studies led us to determine whether the observed apoptotic effects in pancreatic cancer cells dosed with GT3 involved changes in ceramide transport and levels in K-Ras mutated cells as compared to wild type. Here we show that GT3 causes an increase in the levels of certain ceramides at the plasma membrane by the upregulation of enzymes involved in both the *de novo* pathway and the hydrolysis of sphingomyelin, and the modulation of ceramide transporters regardless of K-Ras status. The apoptotic nature of these changes is confirmed by the clustering of death receptor 5 at the membrane and confirming previous observations of the mechanism of action by which GT3 inhibits cell proliferation and survival in pancreatic cancer cells.

## Methods

### Cell lines and culture conditions

MIA PaCa-2 (CRM-CRL-1420), BxPc3 (CRL-1687), and Panc 1 (CRL-1469) cells were obtained from the American Type Culture Collection (Manassas, VA) and were maintained as described before [11]. Human pancreatic ductal epithelial cells (HPDE-E6E7), a generous gift from Dr. Ming-Sound Tsao (Ontario Cancer Institute, Toronto, Ontario, Canada), were cultured in keratinocyte medium (Fisher Scientific, Waltham, MA) as described elsewhere [16]. For immunoblotting experiments, lentiviral transduction, LC/MS and qRT-PCR, cells were seeded on 60 mm plates at high density (~5x10^4^ cells/cm^2^) to obtain confluency in 2–3 days. For immunofluorescence experiments, the cells were seeded on 12mm round cover slips (Fisher Scientific) or 6-mm Transwell-Clear^TM^ filters (Corning Costar) at high density (~5x10^4^ cells/cm^2^) and treated at 70% confluency.

### SDS PAGE and immunoblotting

Cells (70% confluent) were treated with GT3 (Cayman Chemical, Ann Arbor, MI) dissolved in ethanol, at a concentration of 40 μM or dissolution vehicle as a control and incubated for 2, 4, or 6 hours. The cells were rinsed with phosphate-buffered saline and lysed in the plate with buffer (20 mM Imidazole-HCl, pH 6.8, 100 mM KCl, 1 mM MgCl2, 10 mM EGTA, 0.2% (v/v) Triton X-100,) containing phosphatase and protease inhibitors (Sigma Aldrich, St. Louis, MO). The protein concentration of the cell lysates was determined using the Advanced protein assay reagent (Cytoskekleton, Denver, CO). Equal amounts of proteins in cell lysates were separated in 7 or 10% SDS-PAGE. The proteins were transferred to nitrocellulose membranes (Pall Life Sciences, Ann Arbor, MI). Immunoblot procedures were done according the protocol for each antibody. Membranes were probed with primary antibodies against ASM, DEGS1, SPT, Collagen type IV alpha-3-binding protein, also known as ceramide transfer protein CERT, DDIT3 (Abcam, Cambridge, MA), ceramide synthase 6 (Abgent, San Diego, CA), ACAT-related enzyme-2 required for viability, also known as ARV1 (Santa Cruz Biotechnology, Dallas, TX), DR5 (Sigma Aldrich), and caveolin (Cell Signaling Technology, Danvers, MA).

### Lentiviral transduction of ARV1 shRNA

shRNA-expressing lentiviral particles against human ARV1 (NM_022786) were obtained from Sigma Aldrich (5’CCGGGCCAGAAACCTGTAGACAAATCTCGAGATTTGTCTACAGGTTT CTGGCTTTTTG-3’) clone no. TCRN0000107011; MIA PaCa-2 cells were transduced at 70% confluency and treated with GT3 or dissolution vehicle, 48h after lentiviral transduction.

*qRT-PCR for ARV-1 and ceramide enzymes*

To study the levels of ARV1, quantitative RT-PCR was performed on MIA PaCa-2 cells. Total RNA was isolated using Trizol (Invitrogen) according to the manufacturer's instructions and treated with DNase I using the RNeasy Mini Kit and on-column RNase free DNase kit (Qiagen). 1.0 μg RNA was reverse-transcribed with the Super script II Kit (Invitrogen) as recommended. The ARV-1 primers used were: (GCC ACC ACC TCA GGT ATG CTT C) and (GTG CAA AGC TCA GGC CTA CAG AC).

### Immunofluorescence

Cells were dosed with 40μM GT3 or ethanol as a control and processed for immunofluorescence as described before [17]. Briefly, cells were rinsed with phosphate-buffered saline and fixed with 4% p-formaldehyde. Then the cells were rinsed and permeabilized with 0.2% Triton-X100, followed by quenching with NH4Cl. Cells were then incubated with primary antibody in 1% bovine serum albumin at room temperature for one hour. Antibodies against ceramide, death receptor 5 (DR5) (Sigma) and caveolin (Cell Signaling Technology) were used as primary antibodies for immunofluorescence. Cells were then rinsed and incubated for one hour in the dark with secondary antibody conjugated to fluorescent dyes Alexa Fluor 488® and Texas Red® (Molecular Probes, Eugene, OR). DAPI stain was used to visualize nuclei. Cells were then mounted in 10% polyvinyl alcohol, 30% glycerol, 1% n-propyl gallate and SlowFade^TM^ (Molecular Probes). Laser confocal microscopy was performed with a Zeiss LSM 710 confocal microscope (Carl Zeiss MicroImaging GmbH, Germany) in the Imaging Core, Quillen College of Medicine, East Tennessee State University. Cell monolayers were analyzed using a 63 x oil immersion objective. The images were collected using the LSM 710 software (Carl Zeiss Micro Imaging).

### Preparation of cellular samples for LC/MS

All solvents for sample extraction and LC/MS were LC/MS grade (Fisher Scientific), other reagents were purchased from Sigma-Aldrich (St. Louis, MO, USA) or Fisher Scientific. Calibration standards and internal standards were purchased from Avanti Polar Lipid, Inc. (Alabaster, AL). Crude plasma membrane isolation was carried out at 4°C. Briefly, cells were resuspended in lysis buffer (NaHCO3 1mM, NaCO3 0.011mM, CaCl2 1mM, MgCl2 1mM, pH 7.4), and incubated on ice for 20 minutes, followed by homogenization with a Dounce homogenizer. The homogenates were centrifuged at 500 g for 5 minutes; the resulting supernatant was transferred to another microcentrifuge tube, and the remaining pellet containing the nuclear fraction was discarded. An equal volume of 510mM sucrose solution was added to the supernatant, and separated by centrifugation at 20,000g for 30minutes. The supernatant was transferred to an ultracentrifuge tube, and the remaining pellet containing mitochondria, Golgi apparatus and part of the microsome was set aside. The supernatant was ultracentrifuged at 240,000 g for 2 hrs. The resulting supernatant was transferred to another tube as cytosol sample, whereas the crude plasma membrane was settled in the pellet. The pellet was washed with lysis buffer and resuspended with PBS as plasma membrane sample. Samples were frozen at -80 C for later analysis. The extraction of lipids from the plasma membrane was performed in the following manner, 1 ml of methanol containing 20μl of 2μM of each internal standard (C12 ceramides, C12 dihydroceramide, C12 sphingomyelin, C17 sphingosine, C17 sphinganine, C17 sphingosine-1-phosphate, and C17 sphinganine-1-phosphate) were added to 100μl aliquot of sample in a clean glass tube. The mixture was centrifuged at 3,000g for 10 minutes and the supernatant was transferred to a second glass tube and evaporated under a nitrogen stream. The extracted lipids were reconstituted in methanol: acetonitrile (v:v=50:50) and transferred to LC/MS autosampler vials (Waters, Milford, MA) for injection.

### LC/MS

All experiments were carried out on a Waters Xevo TQ MS ACQUITY UPLC system (Waters). The system was controlled by Mass Lynx Software version 4. 1. The sample was maintained at 4°C in the autosampler and was loaded onto a Waters ACQUITY UPLC BEH Phenyl column (3 mm inner diameter × 100 mm with 1.7 μm particles), preceded by a 2.1×5 mm guard column containing the same packing. The column was maintained at 40°C throughout analysis. The UPLC flow rate was continuously 300μL/min in a binary gradient mode with the following mobile phase: initial flow conditions were 20% solvent A (H2O, containing 0.2% formic acid and 0.1% ammonium formate) and 80% solvent B (acetonitrile, containing 0.2% formic acid and 0.1% ammonium formate). Solvent B was increased linearly to 95% over a 2 min period and to 98% in the subsequent 6 min. This was followed by a reduction of solvent B to 80% starting at 8.2 min and continuing through 9 min. Ceramides of interest eluted between 4.0 and 7.5 min. Positive ESI-MS/MS mass spectrometry was performed to identify ceramide species. Different species were confirmed by comparing the retention times of experimental compounds with those of authentic standards. Concentrations of ceramides in the samples were quantified by comparing integrated peak areas for those of each ceramide against those of known amounts of purified standards. Loss during extraction was accounted for by adjusting for the recovery of the internal standard added before extraction. Positive ESI-MS/MS was performed using the parameters described under supplementary information.

*Statistical analysis*. Data are represented as the mean ± SE. In all cases, *n* refers to the number of independent experiments. When comparisons were done relative to the control, statistical analyses were run by Student's *t* test, *p* < 0.05 was considered significant. Statistical significance of comparisons between different treatments was assessed using ANOVA (GraphPad Prism 7, La Jolla, CA).

## Results

**Fig. 1:**
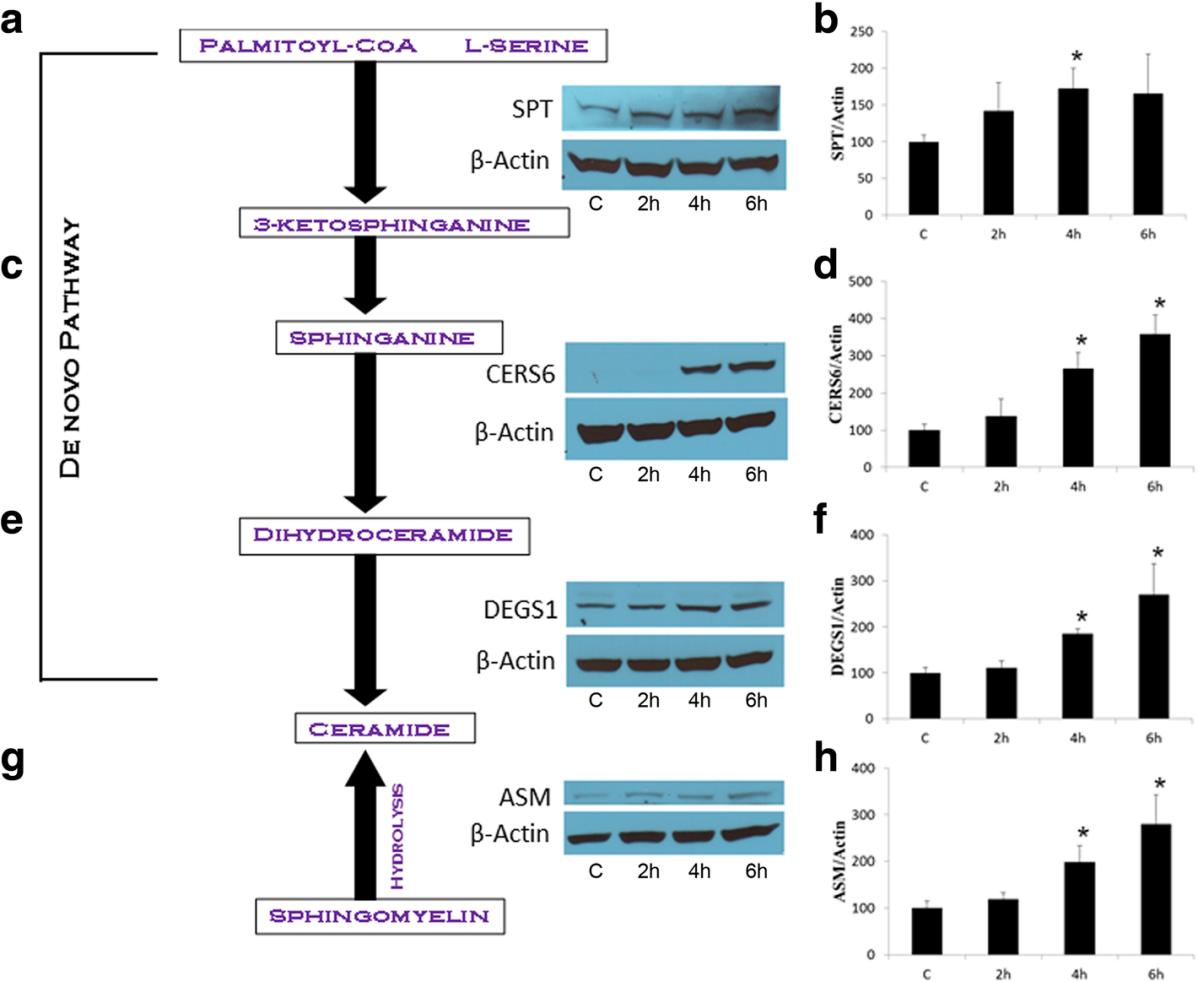
*Time-dependent increase of ceramide synthesis enzymes in GT3 treated MIA-PaCa-2 cells.*Ceramide is produced via the *de novo *pathway (**a**, **c**, **e**) each step is catalized by the following enzymes, SPT(**b**), CER6 (**d**), and  DEGS1(**f**). It is also produced by hydrolysis of sphingomyelin (**g**) by  ASM (**h**). Pancreatic cancer MIA PaCa-2 cells were treated with GT3 at a concentration of 40 μM or dissolution vehicle as a control for different time periods (2, 4, 6 h). Cell lysates were analyzed by immunoblot. Membranes were incubated with antibodies against SPT(**b**), CERS6 (**d**), DEGS1(**f**), and ASM(**h**). The membranes were reprobed with actin as a loading control. All assays were conducted at least three times and blots shown are representative of the results obtained. For quantification band densities from the treatment conditions identified by the lane labels, were calculated as percentages of the value for the treated, untreated cells (100%), and shown averages ± standard deviations from three independent experiments (*p<0.05)

**Fig. 2:**
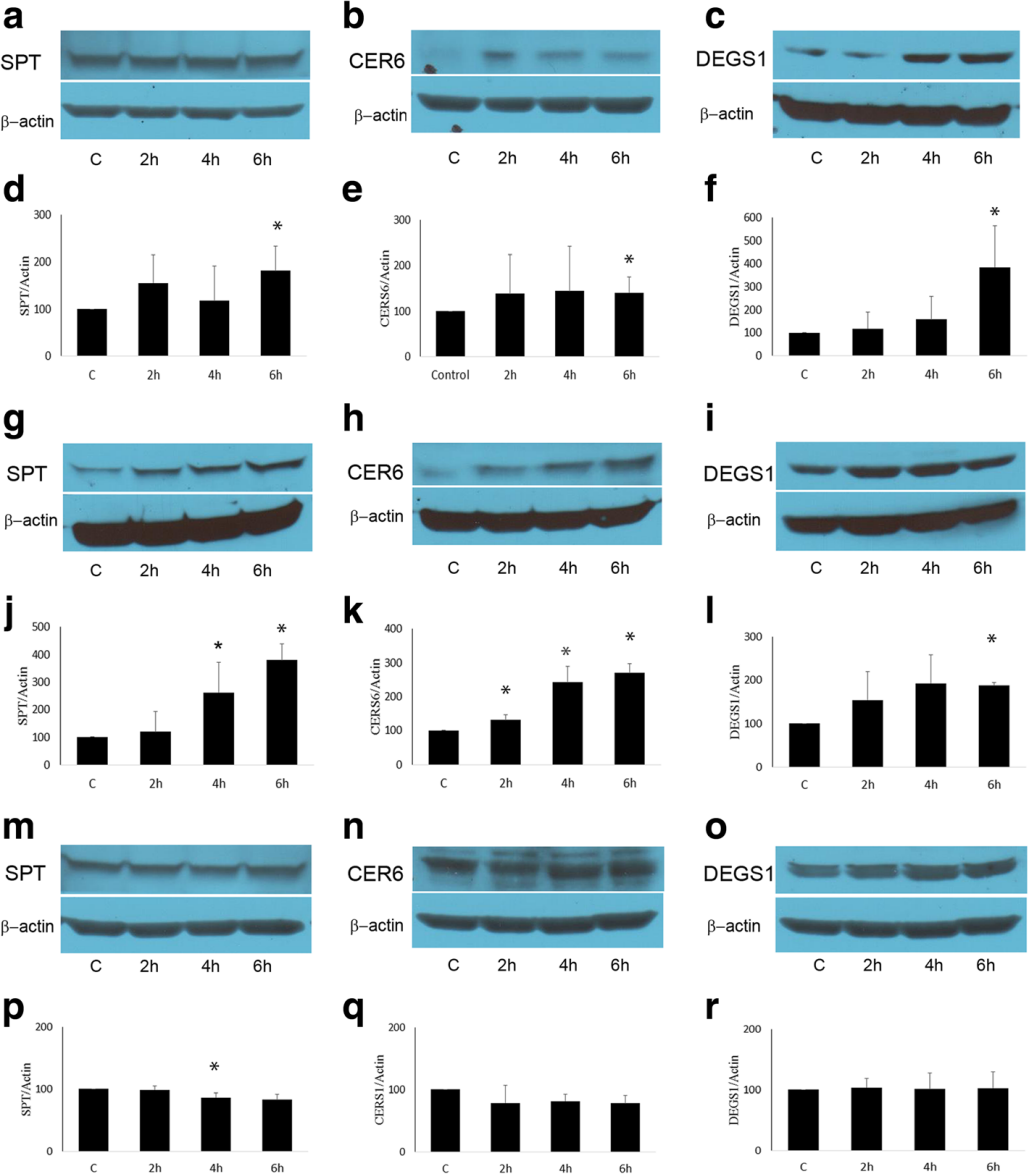
*Time-dependent analysis of enzymes involved in ceramide synthesis in GT3 treated BxPC3, Panc 1 and HPDE-E6E7 cells.* Pancreatic cancer cells BxPC3(**a**-**f**), Panc 1 (**g**-**l**), and non-cancerous ductal pancreatic cells HPDE-E6E7 (**m**-**r**) were treated with GT3 at a concentration of 40 μM or dissolution vehicle as a control for different time periods (2, 4, 6 h). Cell lysates were analyzed by immunoblot. Membranes were incubated with antibodies against SPT, CERS6, and  DEGS1. The membranes were reprobed with actin as a loading control (located below each set of enzyme immunoblots, as in Figure 1). For quantification band densities from the treatment conditions identified by the lane labels, were calculated as percentages of the value for the treated, untreated cells (100%). Data shown of representative experiments, (mean ± SE, *n* = 3) *p<0.05, **p<0.01, significant difference between control and GT3 treated cells analyzed at different time points

*Upregulation of ceramide via the de novo pathway and the hydrolysis of sphingomyelin by GT3 in pancreatic cancer cells regardless of their Ras status.* Previous studies had shown that apoptosis is induced by GT3 in both wild type and mutated K-Ras pancreatic cancer cell lines via a mechanism that involves disruption of signaling of receptor tyrosine kinase ErbB2. To test whether changes in ceramide expression levels occur, we probed the ceramide *de novo* synthesis pathway by analyzing the expression of enzymes SPT, CERS-6, DEGS1 at 2, 4, and 6 hours after dosing with GT3, K-Ras mutated MIA PaCa-2 (Fig. 1a-f) and Panc 1 (Fig. 2a-f), and wild type BxPC3 pancreatic cancer cells (Fig. 2g-l). The expression levels of all enzymes tested increased in a time-dependent manner in MIA PaCa-2, Panc 1, and BxPC3 pancreatic cancer cells. A 1.5 fold increase of SPT is apparent 2 hours after dosing MIA PaCa -2 cells with GT3 as compared to the control (Fig. 1a and b). Similarly, DEGS1 levels increase in all cell lines tested with up to 3.0 fold increase in MIA PaCa-2 cells (Fig. 1e and f). A robust increase in the CERS6 levels at 4 and 6 hours after treatment with GT3 also support the activation of this pathway (Fig. 1c and d). Many recent studies have demonstrated the central role of ASM in the apoptotic process via the formation of ceramide-enriched membrane domains, specifically by gamma radiation [18, 19], UV light [20, 21], and chemotherapeutic agents such as cisplatin [22] and doxorubicin [23]. To determine whether GT3 activates the hydrolysis of sphingomyelin, we probed the levels of ASM. Our results indicate that GT3 may also favor the hydrolysis of sphingomyelin to produce ceramide by increasing the concentration of ASM by 3.4 fold in MIA PaCa-2 cells after treatment with GT3 (Fig. 1g and h). A similar trend on the levels of ASM was observed in Panc 1 and BxPC3 pancreatic cancer cells (results not shown). These results suggest that the activation of ceramide synthesis by GT3 is likely to increase the ceramide levels in the treated cells. Conversely, in non-cancerous human pancreatic ductal epithelial cells (HPDE-E6E7), GT3 has no significant effects on any of the enzymes tested, as compared to the control (Fig. 2m-r).

**Fig. 3:**
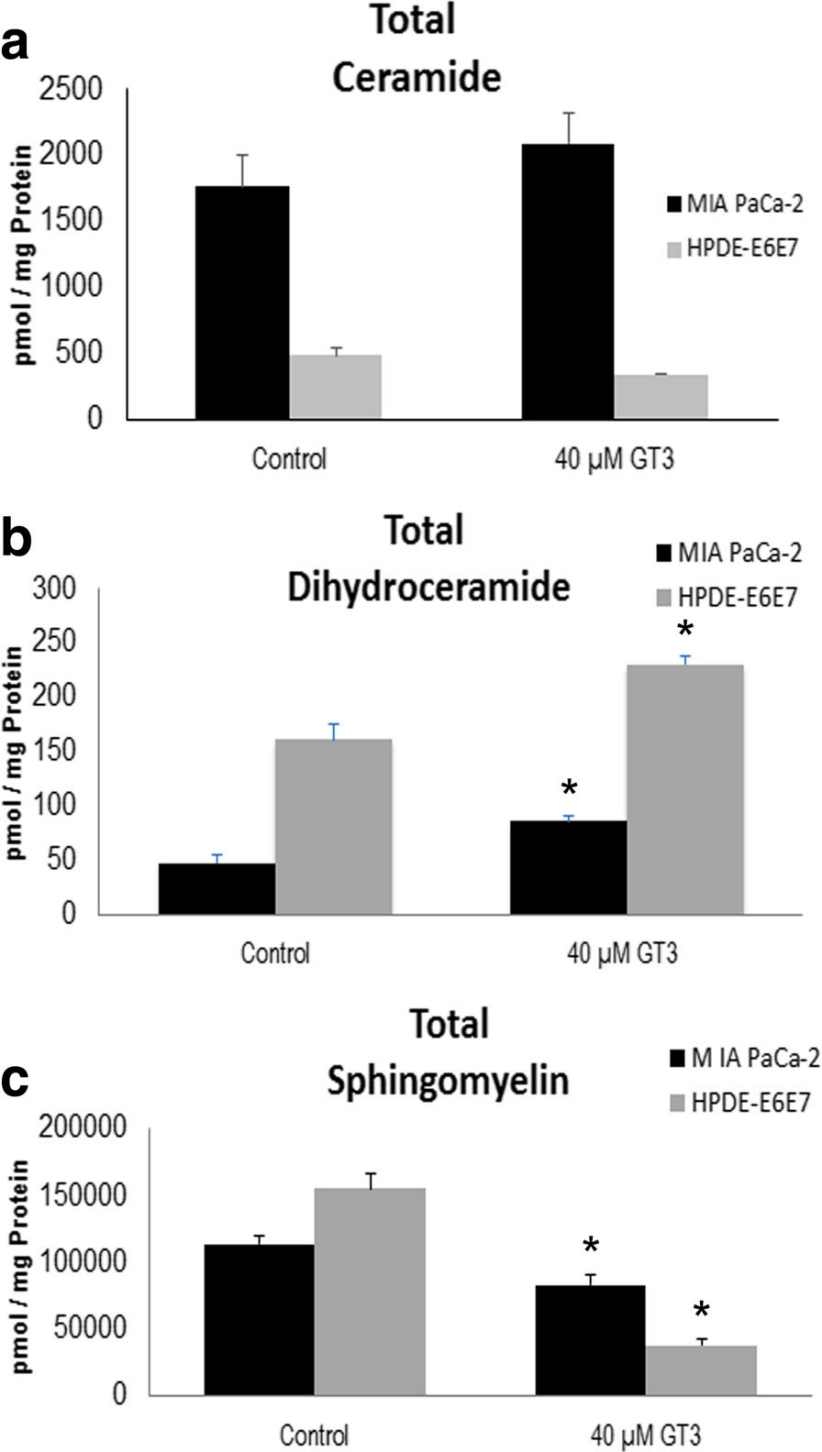
*Analysis of the effect of GT3 treatment on cellular levels of ceramide,* *dihydroceramide, and sphingomyelin.* A. LC/MS analysis of sphingolipids present in the plasma membrane for ceramides (**a**), dihydroceramides (**b**), and sphingomyelin (**c**), in MIA Paca-2, and HPDE-E6E7 cells treated with dissolution vehicle (control) or 40 μMGT3. Results are shown as pmol of each indicated sphingolipid / mg of protein in the sample analyzed. The values shown are the averages ± standard deviations obtained from three independent experiments. * *p* <0.05, significant difference between control and GT3 treated cells

*Pancreatic cancer cells MIA PaCa-2 have higher total ceramide content than non-malignant pancreatic HPDE-E6E7 epithelial cells and GT3 cause a further increase in ceramides and dihydroceramides in MIA PaCa-2 cells.* Ceramide is an important regulator of cellular homeostasis, involved in signaling pathways of apoptosis, senescence, and differentiation [24]. However, its expression levels are normally low and upregulation of ceramide concentration is tightly controlled. Increased levels of ceramides are known to cause apoptosis and are also involved in oncogenesis. To test whether pancreatic cancer MIA PaCa-2 cells have higher ceramide levels than epithelial pancreatic HPDE-E6E7 cells, the total content of ceramides was analyzed by LCMS. The results obtained are in agreement with previous studies in other cancers, and show that MIA PaCa-2 cells have approximately three-fold expression levels than non-malignant (HPDE-E6E7) pancreatic cells (Fig. 3a). Further accumulation of ceramides in the cell membrane may activate their apoptotic function, and support the observations in studies that have shown inhibition of cell viability by GT3. LCMS analysis showed that GT3 cause a further increase of 72.9%±1.82 total membrane ceramides in MIA PaCa-2 cells, confirming the observations on the activation of synthesis pathways of ceramide (Fig. 3b). Dihydroceramides are present at significantly lower concentrations as compared to ceramides in both MIA PaCa-2 and HPDE-E6E7 cells. Unlike ceramides, dihydroceramide levels in the membrane are three fold higher in HPDE-E6E7 than in MIA PaCa-2 cells. These compounds show an increase of 80.5 %±1.16, and 42.97%±3.47 after treatment with GT3 of MIA PaCa-2 and HPDE-E6E7 cells, respectively (Fig. 3b).

*Membrane sphingomyelin is significantly decreased in MIA PaCa-2 and HPDE-E6E7 cells treated with GT3.* Besides the *de novo* pathway, ceramide synthesis can occur via the hydrolysis of sphingomyelin. In order to determine whether there is a significant change in sphingomyelin content, LCMS analysis was conducted on cell lysates of GT3 treated and untreated MIA PaCa-2 and HPDE-E6E7 cells. In untreated cells, non-malignant cells have only slightly higher levels of sphingomyelin than pancreatic cancer cells (Fig. 3c). A significant decrease in membrane sphingomyelin of 26.54%±0.071 in MIA PaCa-2, and 70.05% ±11.17 in HPDE-E6E7 was observed after treatment with GT3, supporting our previous data on the activation of the ceramide synthesis pathway via sphingomyelin hydrolysis (Fig. 3c).

**Fig. 4:**
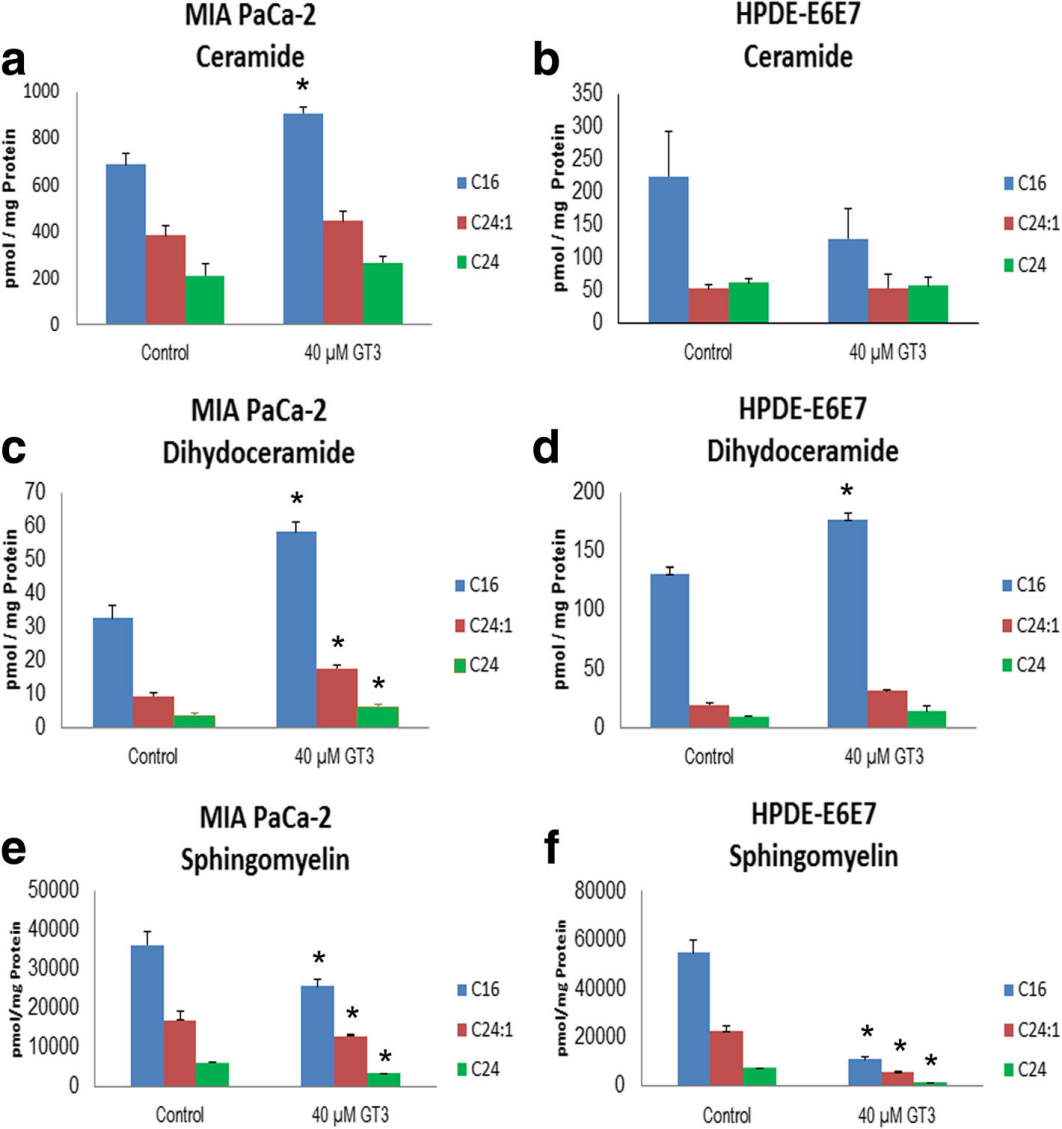
*Analysis of the effect of GT3 treatment on the expression levels of ceramides C16, C24:1 and C24.* A. LC/MS of C16, C24:1 and C24 ceramides in MIA PaCa-2 cells (**a**), and HPDE-E6E7(**b**) dihydroceramides (**c** and **d**), and sphingomyelin (**e** and **f** ) treated with dissolution vehicle (control) or 40μM GT3. Results are shown as pmol of each indicated sphingolipid/mg of protein in the sample analyzed. The values shown are the averages ± standard deviations obtained from three independent experiments. * *p* <0.05, significant difference between control and GT3 treated cells

*GT3 cause an increase in membrane ceramides and dihydroceramides C16, C24:1, and C24 and a decrease in sphingomyelins in MIA PaCa-2 cells* Ceramides with a different fatty acid chain length, have different cellular functions. It has been previously reported that increase in the levels of C16, C24:1 and C24 ceramides, can induce cell death in lymphoma cells after treatment with cannabinoids [25], and the specific upregulation of C16 ceramide in leukemia and colon cancer cells can cause apoptosis [26]. To test whether membrane C16, C24:1, and C24 ceramides are altered by GT3 treatment of MIA PaCa-2 and HPDE E6E7 cells, we analyzed these compounds by LC/MS. Our data show that in untreated cells, C16 is present at 2.7-fold higher levels in MIA PaCa-2 than in HPDE E6E7 cells. Similarly, C24:1, and C24 ceramides, are also present at 7.5-fold and 3.5 fold higher levels in MIA PaCa-2 than in HPDE E6E7 cells. Treatment with GT3 produce a significant change only in C16 ceramide, with an increase of 32.36% as compared to the control (Fig. 4a). Conversely, in non-malignant HPDE-E6E7 cells, the only membrane ceramide that is observed to change significantly is also C16 with a 41.47% decrease as compared to the control (Fig. 4b). Dihydroceramides are present at significantly lower concentrations than ceramides in MIA PaCa-2 cells. However, these compounds may contribute to the total content of cellular ceramides. Our results show that GT3 cause an increase in dihydroceramides of 79.08% 87.34%, and 83.44% for C16, C24:1, and C24, respectively as compared to the control (Fig. 4c). HPDE-E6E7 cells have higher concentrations of dihydroceramides at the cellular membrane than MIA PaCa-2 cancer cells. Our results show that only C16 display a significant increase of 59.52% (Fig. 4d). All sphingomyelins tested displayed a significant decrease in MIA PaCa-2 cells after treatment with GT3. LCMS analysis of sphingomyelin in MIA PaCa-2 cells treated with GT3 showed a significant decrease of 29. 2% (C16), 24.44 (C24:1), and 45.0% (C24). (Fig. 4e). Similarly to sphingomyelins in MIA PaCa-2 cells, GT3 cause a significant decrease in HPDE-E6E7 -E6E7 cells of 79.5% (C16), 74.8% (C24:1), and 79.3% (C24) (Fig. 4f).

*Ceramide transporter CERT is downregulated and ER-localized sterol transport protein ARV1 is upregulated by GT3 in MIA PaCa-2, BxPC3, and Panc1 pancreatic cancer cells.*

**Fig. 5:**
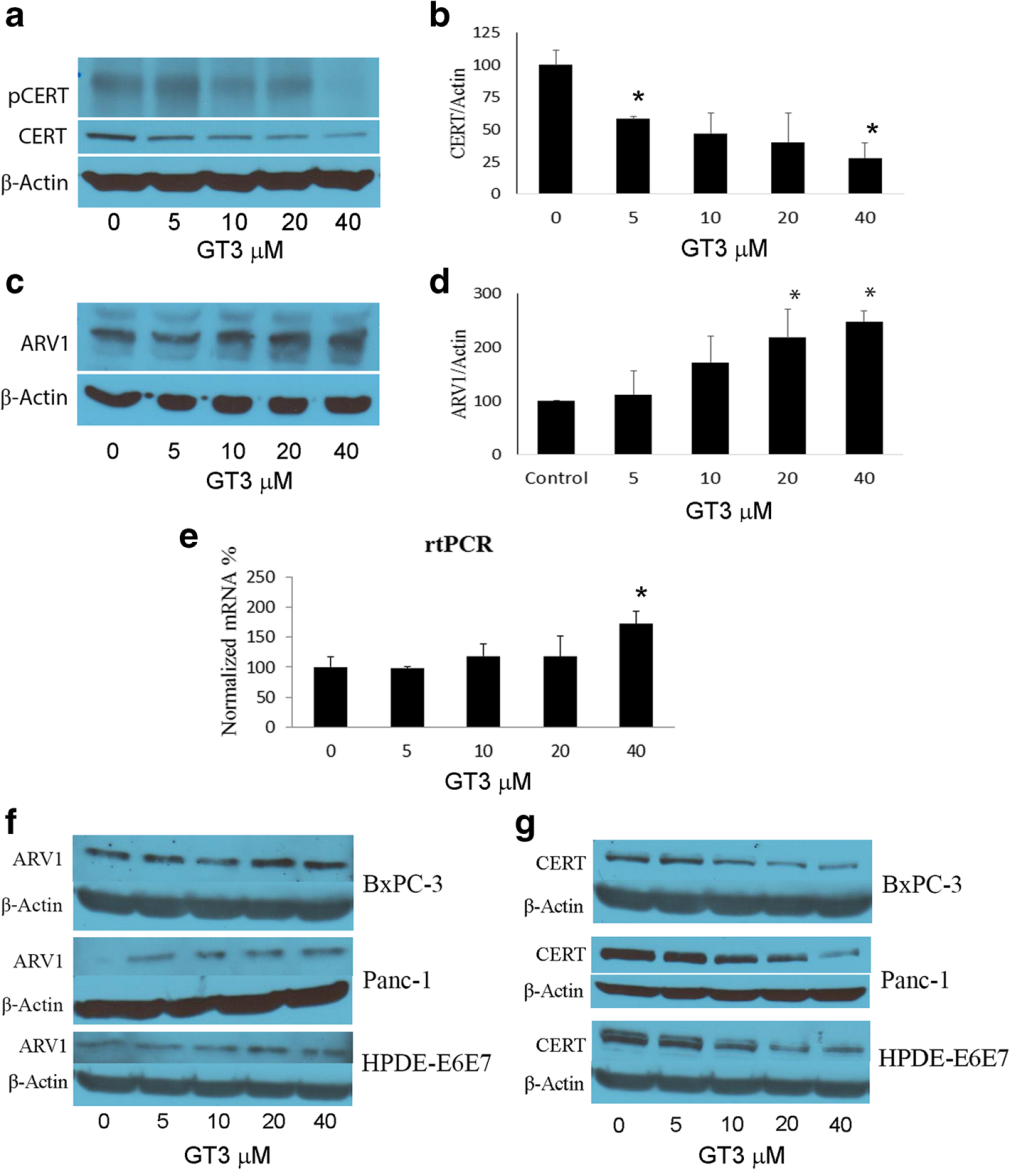
*Downregulation of ceramide transporter CERT and upregulation of transport protein, ARV-1 in MIA PaCa-2, BxPC3, Panc 1 and HPDE-E6E7 cells treated with GT3.* Pancreatic cancer cells MIA PaCa-2, BxPC3, and Panc 1, as well as HPDE-E6E7cells were treated for 4 hours with increasing concentrations of GT3 over a dose range from 0-40μM and probed for CERT and its activated form (**a** and **b** for MIA PaCa-2 assays, and **g** for BxPC3, Panc1, and HPDE-E6E7), ARV-1 (**c** and **d** for MIA PaCa-2 assays and **f** for BxPC3, Panc1, and HPDE-E6E7), and analyzed by western immunoblot. The membranes were reprobed with actin as a loading control. Quantitative RT-PCR using ARV-1 primers was performed on MIA PaCa-2 cells treated with increasing concentrations of GT3 in the same manner as described above (**e**). All assays were conducted in triplicate and blots shown are representative of the results obtained. For quantification, (graphs) band densities from the treatment conditions identified by the lane labels, were calculated as percentages of the value for the treated, untreated cells (100%). Data shown of representative experiments, (mean ± SE, *n* = 3). * *p* <0.05, significant difference between control and GT3 treated cells at different concentrations

To determine whether the activation of the pathways of ceramide synthesis cause an effect on the expression levels of ceramide transporters, we probed ceramide transport protein CERT and ARV-1. The function of the ceramide transport protein CERT, is to transport newly synthesized ceramide from the ER to the Golgi [27], where the latter is hydrolyzed to sphingomyelin by sphingomyelinase [28]. To determine whether treatment of MIA PaCa-2, BxPC3, and Panc 1 cancer cells with GT3 favor the transport of newly synthesized ceramide to the Golgi, the levels of CERT and its phosphorylated form were probed by immunoblot. As shown in Fig. 5a and b, there is a significant decrease of both, the activated form and total CERT levels in GT3 treated MIA PaCa-2 cells as compared to the control. We also probed ARV1, a sterol/ceramide transport protein that interacts with genes involved in GPI anchor synthesis; it has been shown that GPI assembly is required for ceramide transport from the ER [29]. As shown in Fig. 5c, d and f, ARV-1 displays a dose-dependent increase in expression levels in MIA PaCa-2, BxPC3 and Panc 1 cells treated with GT3 at 5, 10, 20, and 40μM, with ARV-1 expression levels reaching a 3-fold increase at the highest concentration tested in MIA PaCa-2 cells. A similar trend was observed in BxPC3 and Panc 1 cells, suggesting a decrease in sphingomyelin biosynthesis from newly synthesized ceramide (Fig. 5g).This trend is also evident in rtPCR analysis suggesting that GT3 effect on ARV-1 may occur at the transcription level (Fig. 5e).

**Fig. 6:**
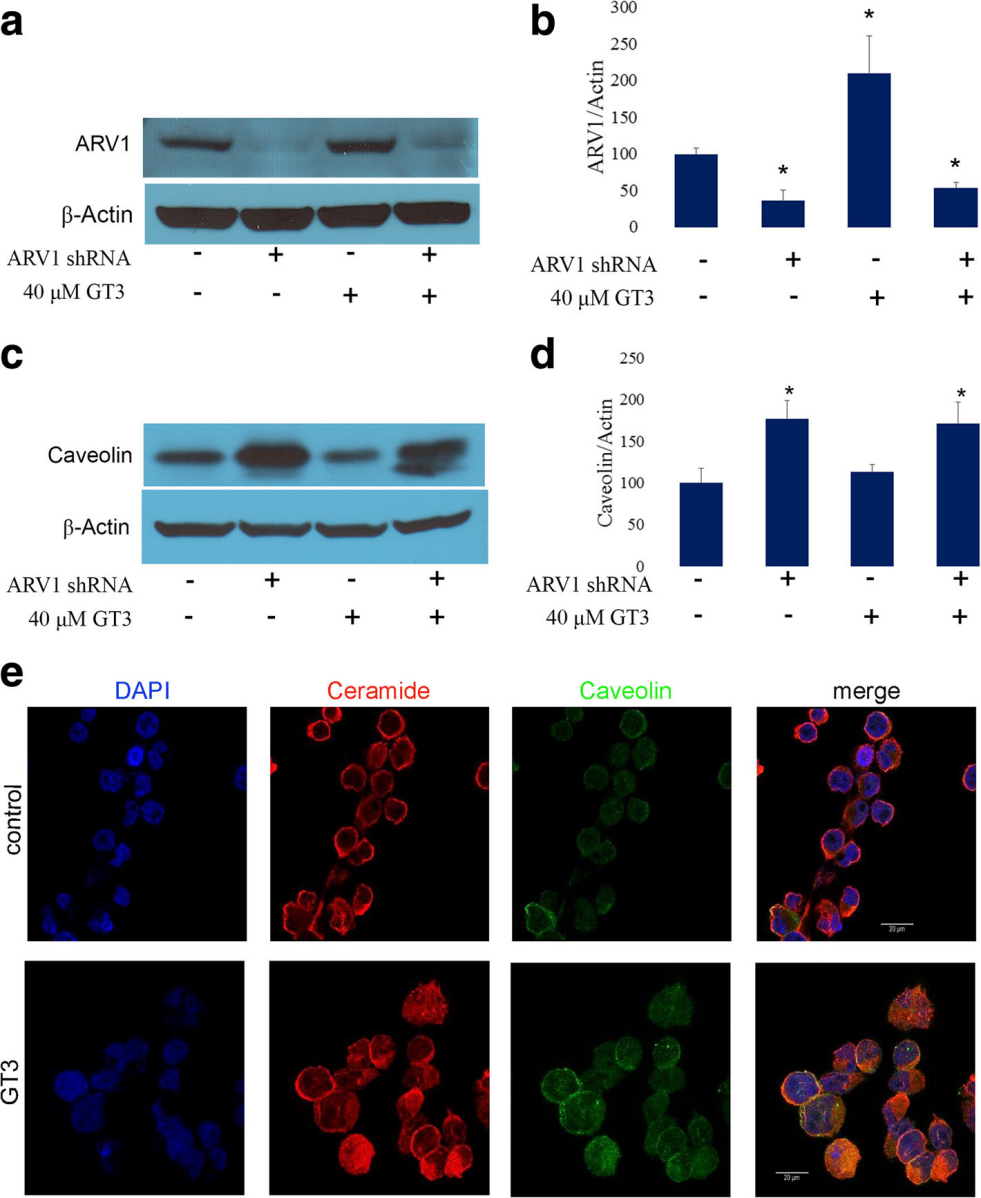
*GT3 effect on ARV-1 knockdown MIA PaCa-2 cells.* MIA PaCa-2 cells were transduced with ARV-1 shRNA lentiviral particles, and then treated with 40μM GT3 or dissolution vehicle (control). Cell lysates were analyzed by immunoblot with antibodies against ARV-1 (**a**,**b**) caveo lin (**c**,**d**). The membranes were reprobed with actin as a loading control. Data shown of representative experiments, (mean ± SE, *n* = 3). * *p* <0.05, significant difference between control and treated cells in different conditions (One-way ANOVA with Bonferroni’s test). **e**.  Expression and localization of ceramide and caveolin in ARV-1 knockdown MIA PaCa-2 cells treated with vehicle (control) or 40μM GT3, and processed for immunofluorescence. Single confocal sections are shown in the x-y plane, ceramide (red channel), caveolin (green channel), and DAPI (blue channel)

*GT3 treatment of ARV-1 inhibited MIA PaCa-2 cells causes upregulation of the expression levels of caveolin-1.* To test whether the effect of GT3 on ARV-1 occurs mainly at the messenger level, protein expression levels were analyzed by immunoblot in MIA PaCa-2 cells transduced with ARV-1 shRNA and treated with GT3. As shown in Fig. 6a and b, ARV-1 is effectively downregulated by ARV-1 shRNA (Fig. 6a and b). GT3 has no significant effect on the expression of ARV-1 (Fig. 6a and b). One of the functions of ARV-1 is to regulate sterol trafficking and plasma membrane structure. It is also known that ceramide is present in caveolae in cancer cells. To test whether GT3 has an effect on caveolin-1, we probed the expression levels of this protein in the presence and in the absence of ARV-1 in MIA PaCa-2 cells. Data shown in Fig. 6c and d suggest that in the presence of ARV-1, there is no significant change in the expression levels of this molecule. However, substantial upregulation is observed in the absence of ARV-1 regardless of GT3 treatment. Caveolin-1 is present in caveolae and is a major component of the vesicular transport system of the trans-Golgi network [27]. To study the role of ARV-1 in the localization of ceramide and caveolin in GT3 treated cells, ARV-1 shRNA transduced cells were processed for immunofluorescence. In the absence of ARV-1, caveolin (green channel) and ceramide (red channel) are localized at the plasma membrane and intracellularly in GT3 treated cells (Fig. 6e).

**Fig. 7:**
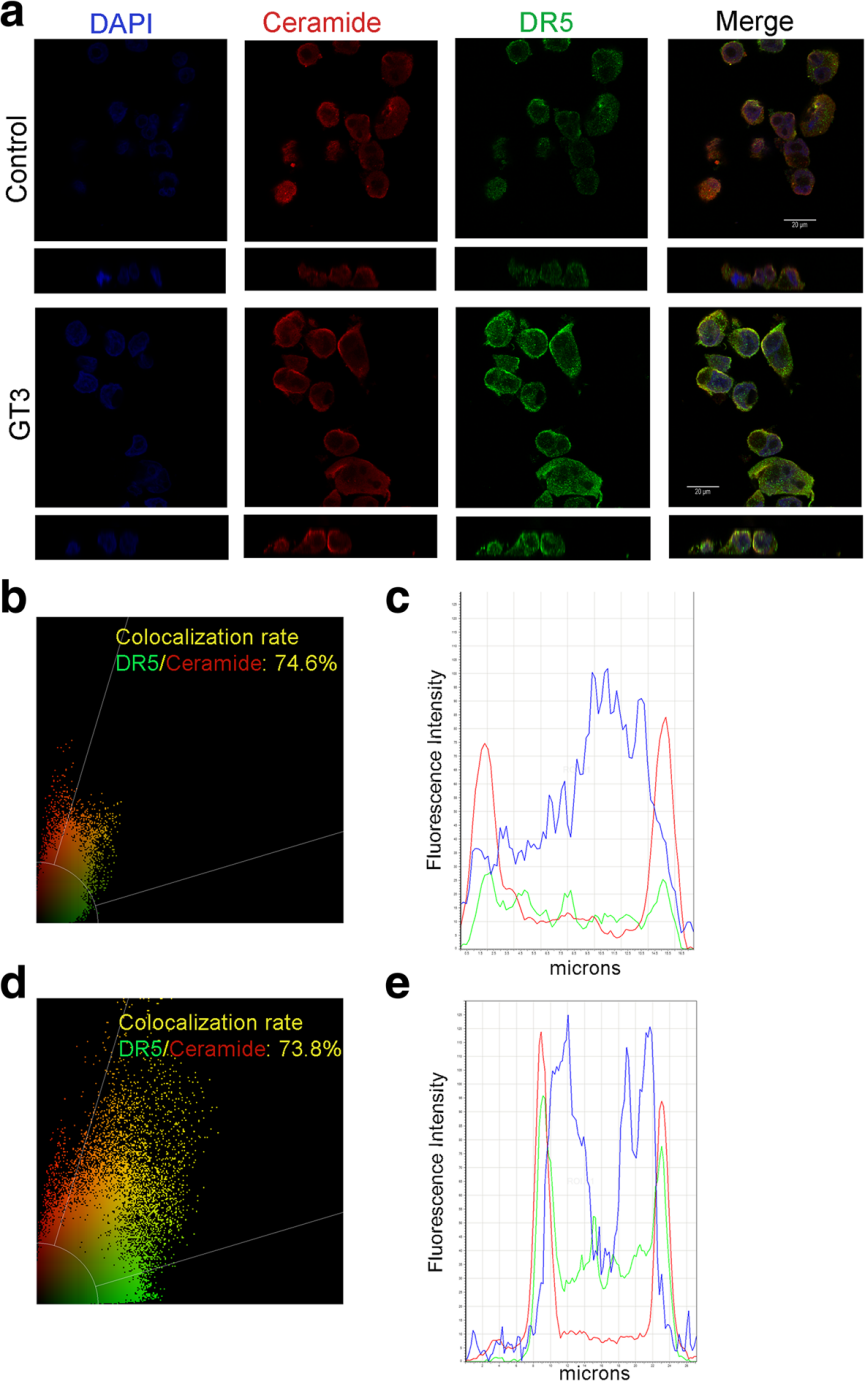
*Colocalization analysis of ceramide and DR5 after GT3 treatment in MIA PaCa-2 cells.* MIA PaCa-2 cells were treated with dissolution vehicle (**a** top panels ) or 40μM GT3 (**a** bottom panels), fixed, and processed for immunofluorescence. Single confocal sections shown in the x-y plane, and 3D reconstructions of the confocal stack in the x–z plane perpendicular to the monolayer, apical side up, show localization of DR5 (green channel), ceramide (red channel), and nuclei using DAPI (blue channel). Intensity correlation analysis of 14 fields, each with an average of 12.5 cells from at least three independent experiments, using the Leica Software were run for control cells (**b**) and (**c**) and treated cells (**d**) and were plotted for both sets of cell populations. The values shown are the averages ± standard deviations obtained from at least three independent experiments. Images are representative of fields analyzed. Graphs illustrating the quantification of relative fluorescence for control cells (**c**) and treated cells (**e**) show the merge for ceramide and DR5 channels. Images shown are representative of the fields observed

**Fig. 8:**
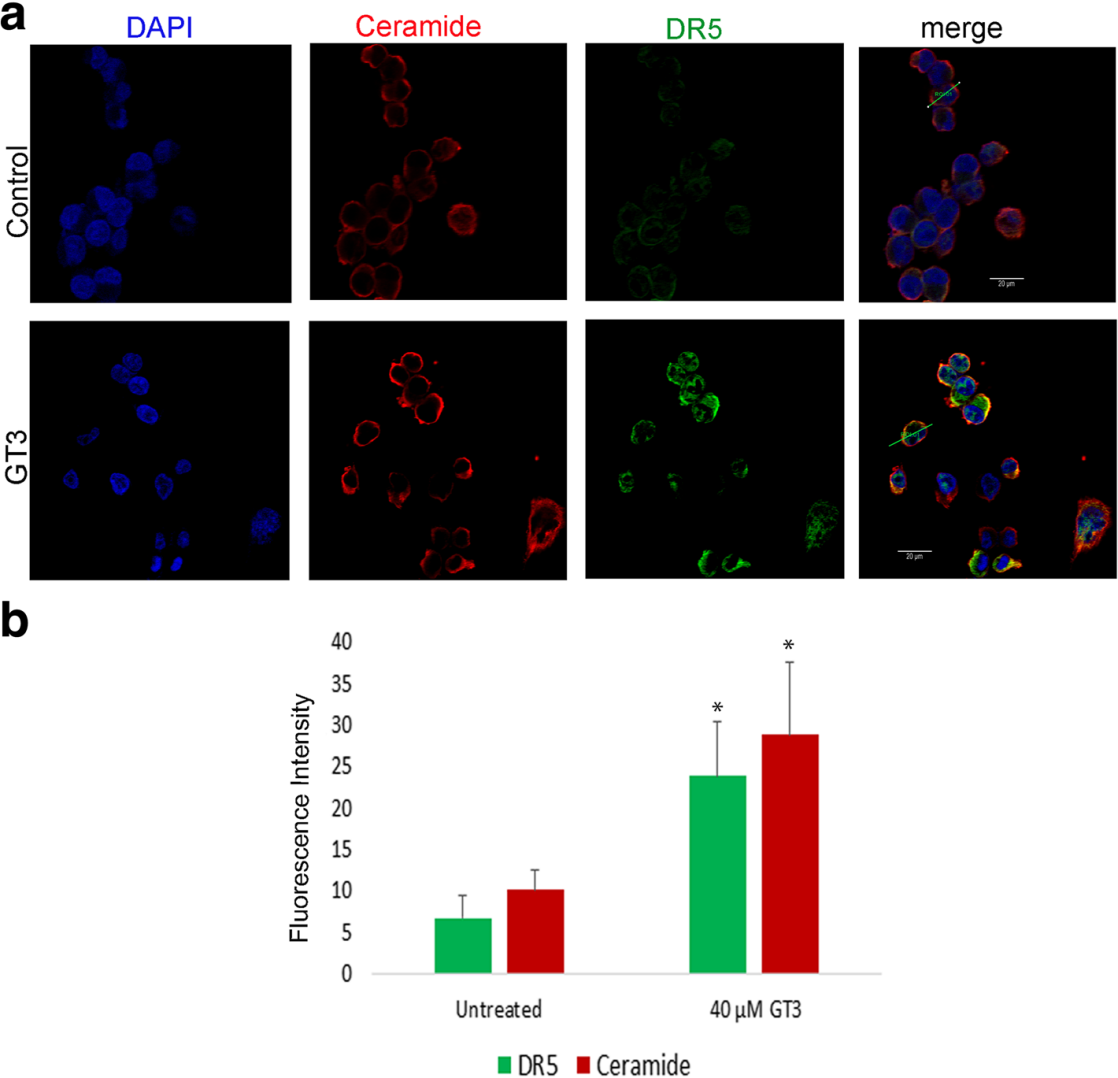
Fluorescence intensity analysis of ceramide and DR5 after GT3 treatment in MIA PaCa-2 *cells.* **a** MIA PaCa-2 cells were treated with dissolution vehicle (A, top panels ) or 40μM GT3 (A, bottom panels), fixed, and processed for immunofluorescence. Single confocal sections shown in the x-y plane, show localization of DR5 (green channel), ceramide (red channel), and nuclei using DAPI (blue channel). **b** The fluorescence intensities along the white lines indicated in the merged pictures were quantified using the line scan application of Leica software. Data obtained from line scans drawn through the cell were plotted in a graph and expressed as mean ±SE, *n*= 50 cells, from three independent experiments.*p < 0.05

*Ceramide-rich caveolae favor clustering of DR5 in GT3 treated pancreatic cancer cells.* It has been reported that a high presence of ceramide in caveolae in human colon cancer cells, favors apoptosis via clustering of Death Receptor 5 [28]. We have previously shown that GT3 has a potent inhibitory effect on ErbB2 phosphorylation [11]. Since ErbB2 is co-localized with caveolin [30], we probed whether the increase of membrane ceramides and the onset of apoptosis by GT3 include the clustering of DR5, by determining the presence and localization of the receptor via immunofluorescence. In untreated and treated cells, ceramide and DR5 are localized at the membrane, red and green channels respectively, Fig. 7a xy and xz planes. Intensity correlation analysis of 14 fields, each with an average of 12.5 cells, showed no significant change with a percentage of colocalization of 74.6% in untreated cells (Fig. 7b) and 73.8% in treated cells (Fig. 7d). However, there was a substantial increase in fluorescence intensity on treated cells, of 3-fold for DR5, and 1.5-fold for ceramide (Fig. 7e) as compared to the control (Fig. 7c). To confirm these observations, analysis of mean intensities of the red and green channels was conducted in these cells (Fig. 8a) GT3 treated cells showed a significant increase in intensities of both channels as compared to untreated cells (Fig. 8b). These results suggest that the activation of apoptosis by GT3 in pancreatic cancer cells may include the participation of DR5 and an increase of ceramide expression levels at the cell membrane suggesting the presence of ceramide micro domains that have been described previously [31].

## Discussion

Our previous work indicated that GT3 has potent anti-proliferative activity in both k-Ras wild type and k-Ras mutated pancreatic cancer cells, but not on non-cancerous pancreatic duct epithelial HPDE-E6E7 cells. The mechanism of action included inhibitory effects on transcription factor NF-κB [12], ErbB2, and the PI3K, MAPK, and HMG CoA reductase pathways, as well as induction of apoptotic signaling through Foxo3 and GSK-3β [11]. However, the coordination of these multi-pronged effects is not completely understood. The results presented in this study suggest that the modulation of expression and localization of ceramide is a relevant factor contributing to the previously observed effects on cell signaling pathways and cell survival. The increase of expression of ASM, responsible for the production of ceramide from sphingomyelin, lends support to this notion. ASM localizes in sphingolipid and cholesterol-enriched membrane domains [32], and the ceramide molecules thus generated, freely associate with each other forming strongly stabilized [33] ceramide-enriched membrane domains [25] by the selective displacement of cholesterol [9]. These changes in membrane composition alter the relatively rapid lateral diffusion characteristic of cholesterol-rich liquid-ordered state [26], and cause destabilization in the structure and function of membrane domains where ErbB2 and other signaling molecules are known to be localized. We had previously shown that cholesterol depletion result in the loss of ErbB2 activation and subsequent inhibition of the ERK pathway [17] It has also been shown that ceramide can cause apoptosis via inactivation of the Ras-Raf/MEK pathway [34]. Thus the exclusion of cholesterol from the plasma membrane, and the downregulation of cholesterol synthesis through inhibition of the HMG CoA reductase pathway by GT3, are likely contributors to the profound changes at the cell membrane imposed by the upregulation of ceramide synthesis. Furthermore, ASM activity also leads to cholesterol displacement from the plasma membrane [35–38] supporting a shift in membrane composition and structure, and subsequently altering signaling function. The resulting ceramide-enriched membrane domains [31], may then favor the presence and clustering of death receptors [28, 39], in agreement with our observation of DR5 localization and clustering at the plasma membrane. These events are required for the formation of death-inducing signaling complexes [40, 41] and are indicative of the activation of the extrinsic apoptotic (JNK) pathway [42], which lend support to our previous results on the upregulation and activation of c-JUN and caspase 8 by GT3 [11]. The role of ceramide in the activation of the JNK pathway and initiation of apoptosis has been demonstrated in several studies [43, 44]. Additionally, DR5 is a component of the TRAIL apoptotic signaling, important for its selective toxicity towards tumor cells [45] and its relation to resistance and sensitization to chemotherapy agents [46]. Our results suggest that GT3 cytotoxic effects carried out via various signaling molecules, are facilitated by the upregulation of expression levels of enzymes involved in the synthesis of ceramide and the subsequent shift in cellular membrane composition and function. However, this set of finely orchestrated events that may obviate the aberrant survival mechanisms present in pancreatic cancer cells, would not be functional without the upregulation of ARV-1 [47] and downregulation of CERT. ARV-1 is involved in sterol transport [48] as well as sphingolipid [49] and glycosylphosphatidyl- inositol [29] biosynthesis. Thus suggesting that GT3 may exert an effect on multiple targets related to control of transcription and molecular synthesis, as well as lipid trafficking and distribution. Loss of membrane cholesterol stimulate sphingolipid transport via CERT [36], and changes in membrane composition by the increase in ceramide content, cause displacement of cholesterol [9]. However, GT3 effectively downregulates CERT, thus causing inhibition of sphingomyelin synthesis in the Golgi from ceramide by diminishing the transport of newly produced ceramide from the ER [50, 51]. Additionally, upregulation of sterol transporter ARV-1, and ceramide synthesis enzymes cause redistribution and upregulation of ceramide at the cellular membrane strongly promoting a profound change in membrane composition and function. Thus, GT3 displays remarkable efficacy in inhibiting the survival mechanisms present in pancreatic cancer cells by its direct involvement in multiple pro-apoptotic events. Specifically, the inhibition of the MAPK and PI 3K/AKT pathways affecting proliferation and survival, the activation of GSK3β and Foxo3 translocation to the nucleus causing G1-phase cell cycle arrest, and activation of the JNK apoptotic pathway via upregulation of and clustering of DR5 at the cell membrane, with subsequent phosphorylation of c-JUN and activation of caspases (10). Additionally, the increase of synthesis of ceramides, specifically, C:16 ceramide a pro-apoptotic molecule. Since most of the newly synthesized ceramides in the ER are used in the Golgi for the synthesis of sphingomyelin and glycosphingolipids [52], the inhibition of CERT [50] causes accumulation of ceramides at the ER, favoring cellular stress and apoptosis. Previous studies have shown increased cytotoxic effects by upregulation of ceramide synthesis in cancer cells.

However, tumor cells treated with drugs that target one or two of the pathways, are likely to fail. Eight approaches to raise ceramide levels have been identified, and many studies suggest that a multiple approach is best to attain a successful intervention [53]. This study has shown that GT3 effectively addresses four of these: stimulation of ceramide synthesis by the *de novo* pathway and sphingomyelin hydrolysis, inhibition of glycoceramide and sphingomyelin synthesis from ceramide. The latter is due to the inhibition of CERT and results in the downregulation of the MDR1 [54] suggesting the involvement of ceramide with the multidrug resistance pathway. The mechanism of action of several current chemotherapeutic agents include the upregulation of cellular levels of ceramide. Treatment with daunorubicin activate the *de novo* ceramide synthesis pathway [55], and may like cisplatin, stimulate sphingomyelin hydrolysis (32, 33).

## Conclusion

Ceramide is a recognized target in oncotherapy. However, in order to obviate the effects of abnormal signaling pathways that result in various mechanisms of resistance in cancer cells, it has been determined that activation of several pathways conducive to raising ceramide levels is necessary for successful activation of apoptosis. The concerted effects on ceramide synthesis, localization and transport by GT3 and its effects on membrane receptors may provide important insight into the targeted pathways necessary to lead pancreatic cancer cells to initiate the apoptotic process and guide the design and development of new therapies for the treatment of pancreatic cancer.

## Abbreviations

ARV-1: ACAT-related enzyme-2 required for viability
ASM: acid sphingomyelinase
CERS6: Ceramide synthase 6
CERT: Collagen type IV alpha-3-binding protein, also known as ceramide transfer protein
DEGS1: Delta(4)-desaturase sphingolipid 1
DR5: Death receptor 5
GT3: γ-tocotrienol
SPT: Serine palmitoyl transferase
